# Complete plastome of *Houttuynia cordata* (Saururaceae), a medicinal and edible plant

**DOI:** 10.1080/23802359.2019.1667910

**Published:** 2019-09-24

**Authors:** Lei Jin, Jin Yang, Changkun Liu, Mengling He, Hanjing Yan

**Affiliations:** a College of Traditional Chinese Medicine, Guangdong Pharmaceutical University, Guangzhou, Guangdong, China;; b Key Laboratory for Plant Diversity and Biogeography of East Asia, Kunming Institute of Botany, Chinese Academy of Sciences, Kunming, Yunnan, China;; c School of Life Science, Yunnan University, Kunming, Yunnan, China;; d Key Laboratory of State Administration of Traditional Chinese Medicine for Production & Development of Cantonese Medicinal Materials, Guangzhou, Guangdong, China

**Keywords:** *Houttuynia cordata*, complete plastome, phylogenetic analysis

## Abstract

The complete plastome of *Houttuynia cordata*, an important medicinal and edible plant, was identified and sequenced in this study. The circular plastome is 160,217 bp in length and consists of a pair of inverted repeats (IRs 26,854 bp each), which is separated by a large single-copy region (LSC, 88,189 bp) and a small single-copy region (SSC, 18,320 bp). It encodes 132 genes, of which 114 are unique genes (80 protein-coding genes, 30 tRNAs, and 4 rRNAs). The phylogenetic analysis strongly reveals the sister group between *H. cordata* and the clade including *Piper kadsura*, *Piper cenocladum*, *Saruma henryi*, and *Asarum sieboldii*.

*Houttuynia cordata* Thunb., a perennial herb belonging to the family Saururaceae, grows in moist and shady places (Shingnaisui et al. 2018). The species is a medicinal plant traditionally used in China, Japan, Korea, and India for the treatment of pneumonia, severe acute respiratory syndrome, muscular sprain, as well as stomach ulcer (Lou et al. 2019). Furthermore, the species is popularly consumed as a healthy vegetable in East Asia (Toda 2005). With the increase of demand and irregular collection, wild *H. cordata* resource has greatly damaged. It is necessary to establish a strategy to conserve for this medicinally and commercially important plant as soon as possible. However, the available genomic resource of *H. cordata* is limited. Here, we report the complete plastome of *H. cordata* using high throughput Illumina sequencing technology.

Samples of *H. cordata* were collected from Tengchong, Yunnan, China (25°25′35′′N, 98°39′07′′E). Voucher specimen (Y. Ji 2017131) was deposited in the Herbarium of Kunming Institute of Botany, Chinese Academy of Sciences (KUN). We used the modified CTAB method (Yang et al. 2014) to extract genomic DNA from silica gel dried leaf tissues at first. Subsequently, the purified DNA was shared by sonication so that fragments of 500 bp length was obtained for constructing a paired-end library. Then the paired-end sequencing was performed using Illumina HiSeq 2000 system at BGI (Wuhan, Hubei, China). Plastome of *Asarum sieboldii* (GenBank Accession No. MG551543) was used as reference sequence and we assembled the plastome following the method described by Jin et al (2018). Finally, the annotation of the plastome was performed in Geneious 10.2.3 (Kearse et al. 2012). The plastome was manually checked for start and stop codons and intron/exon boundaries. The validated complete plastome of *H. cordata* was deposited in the NCBI GenBank database under the accession number MN263890.

The *H. cordata* plastome is 160,217 bp in length and consists of a pair of inverted repeats (IRs, 26,854 bp each), which is separated by a large single-copy region (LSC, 88,189 bp) and a small single-copy region (SSC, 18,320 bp). It encodes 132 genes, of which 114 are unique genes (80 protein-coding genes, 30 tRNAs, and 4 rRNAs). Among unique genes, 9 protein-coding genes (*atp*F, *ndh*A, *ndh*B, *pet*B, *pet*D, *rpl*16, *rpl*2, *rpo*C1, and *rps*12), and 6 tRNAs (*trn*A-UGC, *trn*G-UCC, *trn*I-GAU, *trn*K-UUU, *trn*L-UAA, and *trn*V-UAC) contain one intron, while three protein-coding genes (*ycf*3, *clp*P and *rps*12) have two introns.

To identify the phylogenetic position of *H. cordata*, a maximum-likelihood (ML) (Stamatakis 2014) tree was generated using species within the order Piperales. *Drimys granadensis* was used to root the tree. The phylogenetic analysis reveals the sister group between *H. cordata* and the clade including *Piper kadsura*, *Piper cenocladum*, *Saruma henryi*, *Asarum sieboldii* (Figure 1). Our findings will provide a foundation for further investigation of genetic inheritance and evolution of *H.cordata*.

**Figure 1.**
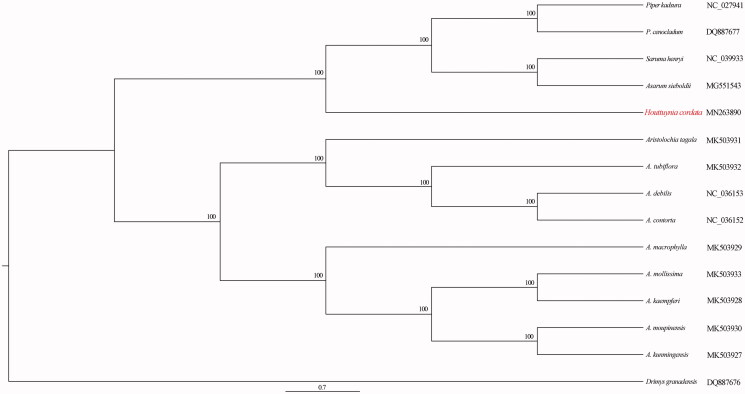
Phylogenetic relationships among Piperales species, based on complete plastomes.
